# Supplementary material for: Dietary Intake of Adult Residents in Luxembourg Taking Part in Two Cross-Sectional Studies—ORISCAV-LUX (2007–2008) and ORISCAV-LUX 2 (2016–2017)
Source: Nutrients. 2021 Dec 7;13(12):4382. doi: 10.3390/nu13124382 (PMC8706514; doi:10.3390/nu13124382)
Supplement: Supplementary file 1 [file nutrients-13-04382-s001.zip › nutrients-1490055-supplementary.pdf]

Supplementary Table S1. Estimated marginal means of participants' total energy, alcohol, and macronutrient intake.

Parameter	Estimated marginal means (95% CI)*		P-value**
	ORISCAV-LUX	ORISCAV-LUX 2	
Total energy intake (kcal/d)	3.349 (3.340, 3.358)	3.375 (3.367, 3.383)	<0.001
Total water (g/d)	3.468 (3.460, 3.475)	3.482 (3.474, 3.489)	0.005
Total protein (g/d)	1.941 (1.932, 1.950)	1.949 (1.941, 1.958)	0.165
Vegetables source protein (g/d)	1.427 (1.418, 1.437)	1.428 (1.419, 1.437)	0.911
Animal source protein (g/d)	1.749 (1.738, 1.760)	1.767 (1.755, 1.778)	0.019
Total Fat (g/d)	1.973 (1.962, 1.983)	2.063 (2.054, 2.072)	<0.001
Saturated fatty acids (g/d)	1.509 (1.498, 1.520)	1.592 (1.582, 1.602)	<0.001
Monounsaturated fatty acids (g/d)	1.597 (1.586, 1.607)	1.673 (1.663, 1.682)	<0.001
Polyunsaturated fatty acids (g/d)	1.179 (1.168, 1.191)	1.323 (1.312, 1.334)	<0.001
Linoleic acid (g/day)	1.092 (1.080, 1.104)	1.240 (1.228, 1.252)	<0.001
Alpha-linoleic acid (g/day)	0.041 (0.029, 0.054)	0.267 (0.254, 0.280)	<0.001
Arachidonic acid (g/day)	-0.812 (-0.825, -0.800)	-0.724 (-0.737, -0.711)	<0.001
Eicosapentaenoic acid (g/day)	-0.937 (-0.958, -0.916)	-0.759 (-0.783, -0.736)	<0.001
Docosapentaenoic acid (g/day)	-1.225 (-1.240, -1.210)	-1.130 (-1.147, -1.113)	<0.001
Docosahexaenoic acid (g/day)	-0.751 (-0.771, -0.731)	-0.584 (-0.605, -0.563)	<0.001
Cholesterol (mg/d)	2.489 (2.478, 2.500)	2.549 (2.538, 2.559)	<0.001
Total carbohydrates (g/day)	2.365 (2.355, 2.374)	2.336 (2.327, 2.345)	<0.001
Simple sugars (g/d)	2.039 (2.028, 2.051)	1.994 (1.983, 2.004)	<0.001
Added sugars (g/d)	1.501 (1.483, 1.519)	1.436 (1.418, 1.454)	<0.001
Starch (g/d)	2.034 (2.023, 2.045)	2.012 (2.001, 2.023)	0.002
Total fiber (g/d)	1.366 (1.356, 1.375)	1.356 (1.347, 1.365)	0.118
Soluble fiber (g/d)	0.675 (0.665, 0.685)	0.662 (0.652, 0.672)	0.046
Alcohol (g/d)	0.654 (0.615, 0.693)	0.788 (0.756, 0.819)	<0.001

* Linear mixed model (based on log-transformed data) adjusted for age and gender.

** Benjamini-Hochberg correction was applied to all p-values: All p-values are displayed after this correction.

Supplementary Table S2. Estimated marginal means of micronutrient intake of participants.

Parameter	Estimated marginal means (95% CI)*		P-value**
	ORISCAV-LUX	ORISCAV-LUX 2	
Vitamin A (µg/day)	2.553 (2.399, 2.707)	2.621 (2.437, 2.804)	<0.001
Beta-carotene (µg/day)	3.601 (3.451, 3.751)	3.649 (3.470, 3.829)	0.013
Vitamin D (µg/day)	0.381 (0.187, 0.575)	0.635 (0.403, 0.867)	<0.001
Vitamin E (mg/day)	1.150 (1.139, 1.161)	1.266 (1.256, 1.276)	<0.001
Vitamin C (mg/day)	2.146 (2.016, 2.276)	2.182 (2.026, 2.338)	0.040
Thiamine (mg/day)	0.189 (0.144, 0.235)	0.193 (0.139, 0.247)	0.726
Riboflavin (mg/day)	0.275 (0.235, 0.314)	0.268 (0.221, 0.315)	0.416
Niacin (mg/day)	1.307 (1.212, 1.402)	1.333 (1.219, 1.446)	0.030
Pantothenic acid (mg/day)	0.722 (0.713, 0.731)	0.767 (0.759, 0.776)	<0.001
Pyridoxine (mg/day)	0.340 (0.331, 0.349)	0.378 (0.369, 0.386)	<0.001
Folate (µg/day)	2.523 (2.458, 2.588)	2.524 (2.447, 2.602)	0.915
Vitamin B12 (µg/day)	0.643 (0.499, 0.787)	0.699 (0.527, 0.872)	<0.001
Calcium (mg/day)	3.022 (3.013, 3.031)	2.970 (2.961, 2.978)	<0.001
Iron (mg/day)	1.141 (1.132, 1.150)	1.151 (1.143, 1.159)	0.067
Iodide (µg/day)	2.159 (2.149, 2.168)	2.191 (2.182, 2.199)	<0.001
Magnesium (mg/day)	2.615 (2.587, 2.644)	2.573 (2.540, 2.606)	<0.001
Potassium (mg/day)	3.540 (3.510, 3.569)	3.543 (3.503, 3.5839	0.726
Phosphorus (mg/day)	3.134 (3.125, 3.143)	3.122 (3.115, 3.130)	0.036
Sodium (mg/day)	3.511 (3.477, 3.545)	3.535 (3.495, 3.750)	0.005

* Linear mixed model (based on log-transformed data) adjusted for age and gender.

** Benjamini-Hochberg correction was applied to all p-values: All p-values are displayed after this correction.

Supplementary Table S3. Estimated marginal means of food groups' intake of participants.

	**Estimated marginal means (95% CI)***		**P-value****
	**ORISCAV-LUX**	**ORISCAV-LUX 2**	
Grains (g/day)	2.282 (2.268, 2.296)	2.065 (2.049, 2.081)	<0.001
Fruits (g/day)	2.413 (2.391, 2.435)	2.420 (2.402, 2.439)	0.561
Vegetables (g/day)	2.406 (2.374, 2.439)	2.293 (2.256, 2.331)	<0.001
Starchy vegetables (g/day)	1.811 (1.599, 2.022)	1.862 (1.609, 2.115)	0.056
Protein rich foods (g/day)	2.189 (2.175, 2.203)	2.314 (2.301, 2.326)	<0.001
Ready to eat and fast foods (g/day)	1.989 (1.796, 2.182)	2.020 (1.788, 2.251)	0.259
Dairy products (g/day)	2.321 (2.299, 2.342)	2.170 (2.146, 2.193)	<0.001
Lipids (fats & oils) (g/day)	1.604 (1.588, 1.621)	1.768 (1.754, 1.783)	<0.001
Sugary products (g/day)	1.659 (1.468, 1.849)	1.578 (1.351, 1.806)	<0.001
- Non-caloric beverages (g/day)	3.129 (3.113, 3.144)	3.197 (3.186, 3.209)	<0.001
- Sugared-sweetened beverages (g/day)	2.214 (1.846, 2.583)	2.245 (1.802, 2.688)	0.543
- Alcoholic beverages (g/day)	1.883 (1.850, 1.916)	1.955 (1.927, 1.983)	<0.001

* Linear mixed model (based on log-transformed data) adjusted for age and gender.

** Benjamini-Hochberg correction was applied to all p-values: All p-values are displayed after this correction.
